# The constant work rate critical power protocol overestimates ramp incremental exercise performance

**DOI:** 10.1007/s00421-016-3491-y

**Published:** 2016-10-27

**Authors:** Matthew I. Black, Andrew M. Jones, James A. Kelly, Stephen J. Bailey, Anni Vanhatalo

**Affiliations:** 1 Sport and Health Sciences, College of Life and Environmental Sciences, University of Exeter, St. Luke’s Campus, Heavitree Road, Exeter, EX1 2LU UK; 2 School of Sport, Exercise and Health Sciences, Loughborough University, Epinal Way, LE11 3TU UK

**Keywords:** Power-duration relationship, Critical power, W′, Performance prediction

## Abstract

**Purpose:**

The parameters of the power-duration relationship (i.e., the critical power, CP, and the curvature constant, W′) may theoretically predict maximal performance capability for exercise above the CP. The CP and Wʹ are associated with the parameters of oxygen uptake (${\dot{\text{V}}}$O_2_) kinetics, which can be altered by manipulation of the work-rate forcing function. We tested the hypothesis that the CP and Wʹ derived from constant work-rate (CWR) prediction trials would overestimate ramp incremental exercise performance.

**Methods:**

Thirty subjects (males, *n* = 28; females, *n* = 2) performed a ramp incremental test, and 3–5 CWR prediction trials for the determination of the CP and Wʹ. Multiple ramp incremental tests and corresponding CP and Wʹ estimates were available for some subjects such that in total 51 ramp test performances were predicted.

**Results:**

The ramp incremental test performance (729 ± 113 s) was overestimated by the CP and Wʹ estimates derived from the best (751 ± 114 s, *P* < 0.05) and worst (749 ± 111 s, *P* < 0.05) individual fits of CWR prediction trial data. The error in the prediction was inversely correlated with the magnitude of the Wʹ for the best (*r* = −0.56, *P* < 0.05) and worst individual fits (*r* = −0.36, *P* < 0.05).

**Conclusions:**

The overestimation of ramp incremental performance suggests that the CP and Wʹ derived from different work-rate forcing functions, thus resulting in different ${\dot{\text{V}}}$O_2_ kinetics, cannot be used interchangeably. The present findings highlight a potential source of error in performance prediction that is of importance to both researchers and applied practitioners.

## Introduction

High-intensity exercise performance is well described by the hyperbolic relationship between power (P) and time, which can be derived from a series of constant work rate (CWR) trials performed until the limit of tolerance (T_lim_) (Hill 1993; Jones et al. 2008; Monod and Scherrer 1965; Moritani et al. 1981; Poole et al. 1988). This hyperbolic relationship is constrained by the capacity and rate at which adenosine triphosphate can be resynthesised via aerobic and anaerobic pathways. The power-asymptote of this hyperbola, termed the critical power (CP), denotes the highest work rate at which a physiological steady-state can be attained and is therefore considered to represent the highest work rate that can be sustained without a significant contribution from anaerobic metabolism (Jones et al. 2008; Poole et al. 1988). The curvature constant (Wʹ) of the power-duration relationship is indicative of a fixed amount of work that can be performed above the CP and is associated with the progressive rise in pulmonary oxygen uptake (${\dot{\text{V}}}$O_2_), and the accumulation of fatigue-related metabolites (i.e., inorganic phosphate, hydrogen ions, interstitial potassium; Allen et al. 2008) until the attainment of maximal O_2_ uptake (${\dot{\text{V}}}$O_2max_) (Poole et al. 1988) and the concomitant achievement of a critical level of intramuscular metabolic perturbation (Vanhatalo et al. 2010). Resolving the parameters of the power-duration relationship, therefore, permits the prediction of exercise performance or tolerance at work rates above the CP according to the equation:

1 $${\text{T}}_{ \lim } = W^{\prime}/({\text{P}} - {\text{CP}})$$

Although the power-duration relationship is conventionally derived from a series of CWR prediction trials, equivalent parameter estimates can also be obtained in a single 3 min all-out test (Burnley et al. 2006; Vanhatalo et al. 2007, 2008). In contrast to CWR exercise, the 3 min all-out test requires subjects to produce their maximal instantaneous power output throughout the test (Burnley et al. 2006; Vanhatalo et al. 2007, 2008). Despite the considerable differences in the work-rate forcing functions between these two testing protocols, similar CP estimates are derived (Simpson et al. 2015; Vanhatalo et al. 2007, 2008). Furthermore, the size of the Wʹ, determined as the work done above CP, has been shown to be similar between ramp incremental, 3 min all-out, and work-matched self-paced time-trial and CWR exercise (Chidnok et al. 2013a). However, Morton et al. (1997) reported a trend for a ~18% lower Wʹ (*P* = 0.07) when power-duration parameters were estimated from a series of ramp incremental prediction trials at different ramp rates relative to CWR prediction trials. The tendency for a smaller Wʹ in ramp compared to CWR protocol indicates that the conventional CWR prediction trial protocol may not accurately predict T_lim_ during ramp incremental exercise.

Performance in ramp incremental exercise, where work rate is increased as a linear function of time (e.g., 1 W every 2 s) can be predicted using a modified version of Eq. 1:

2 $${\text{T}}_{\rm lim} = {\text{CP}}/S + \sqrt{(2{\text{W}}^{\prime}/S)}$$

where *S* represents the ramp slope (e.g., 0.5 W s^−1^) (Morton 1994). The ramp incremental test represents a distinct work-rate forcing function to test the applicability of the CP and W′ estimates derived from CWR prediction trials. During a fast-ramp protocol, the ${\dot{\text{V}}}$O_2_ conforms to quasi-linear first-order kinetics (Whipp et al. 1981; Wilcox et al. 2016), whereas during severe CWR exercise the ${\dot{\text{V}}}$O_2_ kinetics manifests an initial fast (or primary) component followed by delayed, progressive increase in ${\dot{\text{V}}}$O_2_ termed the ‘slow component’ (Burnley and Jones 2007; Poole et al. 1988). The time constant (τ) of the primary component has been inversely correlated with CP and endurance performance (Murgatroyd et al. 2011), while the amplitude of the slow component has been positively correlated with the W′ (Murgatroyd et al. 2011; Vanhatalo et al. 2011). Given that the slow component appears to be almost entirely eradicated (or hidden) during fast-ramp incremental exercise (Wilcox et al. 2016), it is possible that the fixed work capacity indicated by the W′ may not be accessible to the same extent as during severe CWR exercise, consistent with the tendency for lower W′ (Morton et al. 1997).

The purpose of this study, therefore, was to evaluate the accuracy with which ramp incremental exercise performance may be predicted by the power-duration parameters derived from a series of CWR prediction trials. We hypothesized that, due to the differences in ${\dot{\text{V}}}$O_2_ kinetics, the CP and W′ derived from CWR prediction trials would overestimate the ramp incremental test performance using Eq. 2, and that the prediction error would be related to the W′ but not CP.

## Methods

### Overview

This work was a retrospective analysis of data collected during previous research studies for which subjects had performed a ramp incremental test and a series of CWR prediction trials (Black et al. 2015; Kelly et al. 2013; Vanhatalo et al. 2007, 2008). Data were collected in two laboratories (University of Wales Aberystwyth and University of Exeter) and tests were performed after informed consent was provided and following the completion of a health screen questionnaire. Experimental procedures were approved by the local ethics committees. Where available, multiple ramp incremental tests and multiple corresponding parameter estimates (CP and Wʹ) were assessed per subject: 19 males had performed two ramp incremental tests and two sets of prediction trials within the same experimental study, and one male had completed two experimental studies including four ramp incremental tests and four sets of prediction trials. Subjects performed 3–5 prediction trials in all cases (3 trials, 9 cases; 4 trials, 32 cases; 5 trials, 10 cases). In total, 51 data sets, obtained from 30 subjects (males *n* = 28, age, 27 ± 8 years, body mass 75.8 ± 9.8 kg, height 1.79 ± 0.07 m; females *n* = 2, age, 27 ± 4 years, body mass 57.5 ± 0.7 kg, height 1.72 ± 0.03 m) were included in this analysis. Where data had been collected following a supplementation regimen (Kelly et al. 2013), only data from the placebo trials were included in the analysis. The ramp test performance was predicted using parameter estimates derived from CWR prediction trials, where all tests for a given individual were performed within 4 weeks. Subjects were instructed to report to all testing sessions well-hydrated, having avoided strenuous physical activity and caffeine ingestion for 24 and 3 h prior to testing, respectively. Within each study, testing was performed at the same time of day for each subject and laboratory visits were separated by at least 24 h.

## Protocol

### Determination of peak oxygen uptake and GET

All exercise tests were performed using an electronically braked cycle ergometer (Lode Excalibur Sport, Groningen, The Netherlands). The ergometer seat and handlebars were adjusted for comfort, with the cyclists’ own pedals fitted if required, and with the same settings replicated for subsequent tests. The ramp protocol consisted of a period of unloaded pedaling (3 or 4 min), followed by a ramp increase in work rate of 30 W min^−1^ (1 W every 2 s) until volitional exhaustion. Subjects were instructed to maintain their preferred cadence (70–90 rpm) for as long as possible. The test was terminated when the pedal rate fell by more than 10 rpm below their preferred cadence for more than 10 s despite strong verbal encouragement. Power output was recorded to the nearest Watt. The ramp rate (30 W min^−1^) and the end-test power output permitted the determination of T_lim_ to the nearest second. During this and all subsequent tests, breath-by-breath pulmonary gas exchange and ventilation were measured. Subjects wore a nose clip and breathed through a mouthpiece and impeller turbine assembly (Jaeger Triple V, Hoechburg, Germany). The inspired and expired gas volume and concentration signals were continuously sampled at 100 Hz, the latter using paramagnetic (O_2_) and infrared (CO_2_) analysers (Jaeger Oxycon Pro, Hoechburg, Germany) via a capillary line connected to the mouthpiece. These analysers were calibrated before each test with gases of known concentration, and the turbine volume transducer was calibrated using a 3-L syringe (Hans Rudolph, KS). The volume and concentration signals were time-aligned, accounting for the transit delay in capillary gas and analyser rise time relative to the volume signal. Oxygen uptake (${\dot{\text{V}}}$O_2_), carbon dioxide output (${\dot{\text{V}}}$CO_2_) and minute ventilation (${\dot{\text{V}}}$_E_) were calculated using standard formulae (Beaver et al. 1973) and displayed breath-by-breath. Subsequently, the breath-by-breath data were converted to second-by-second data using linear interpolation. The peak ${\dot{\text{V}}}$O_2_ (${\dot{\text{V}}}$O_2peak_) was determined as the highest ${\dot{\text{V}}}$O_2_ over a 30 s period. The data were reduced to 10 s mean values for the estimation of the GET, which was determined as: (1) the first disproportionate increase in ${\dot{\text{V}}}$CO_2_ versus ${\dot{\text{V}}}$O_2_; (2) an increase in minute ventilation (${\dot{\text{V}}}$˙_E_) relative to ${\dot{\text{V}}}$O_2_ with no increase in ${\dot{\text{V}}}$_E_/${\dot{\text{V}}}$CO_2_, and; (3) the first increase in end-tidal O_2_ tension with no fall in end-tidal CO_2_ tension.

### Determination of the power-duration relationship

The CP and Wʹ were estimated from a series of CWR prediction trials performed at different work rates (approximately 60, 70, 80, and 100% ${\dot{\text{V}}}$O_2peak_; where Δ refers to the work rate difference between the GET and the ${\dot{\text{V}}}$O_2peak_). Each prediction trial began with a period of unloaded cycling (3 or 4 min) followed by an abrupt transition to the appropriate work rate. Subjects were instructed to maintain their preferred cadence, which was the same as that chosen during the ramp incremental test, for as long as possible. Trials were terminated when cadence fell by more than 10 rpm below their preferred cadence for more than 5 s or 10 s (for details see; Black et al. 2015; Kelly et al. 2013; Vanhatalo et al. 2007, 2008) despite strong verbal encouragement. Subjects were not informed of the work rate or the performance of any trial until all experimental trials had been completed.

### Data analyses

The CP and Wʹ were estimated using three models: the hyperbolic (P–T_lim_) model, where the work rate is plotted against time (Eq. 1); the linear work-time (W–T_lim_) model, where the work done (W) is plotted against time (Eq. 3); and the linear inverse-of-time (1/T_lim_) model (Eq. 4), where work rate is plotted against the inverse of time.

3 $${\text{W }} = {\text{CP T}}_{ \lim } + {\text{W}}^\prime$$

4 $${\text{P}} = {\text{W}}^\prime \, ( 1/{\text{T}}_{ \lim } ) + {\text{CP}}$$

The standard error of the estimate (SEE) associated with the CP and Wʹ were expressed as coefficients of variation (CV %, i.e., relative to the parameter estimate).

The total error associated with the modelling of the power-duration parameters was calculated as the sum of the CV % associated with the CP and the W′. The sum of the CV % was optimised for each individual by selecting the model (Eqs. 1, 3 or 4) with the smallest total error to produce the “best individual fit” parameter estimates. Similarly, the parameter estimates from a model associated with the largest total error were grouped together to produce the “worst individual fit” parameter estimates. The best fit and worst fit CP and Wʹ derived from the CWR prediction trials were then used to retrospectively calculate T_lim_ during the ramp incremental exercise test using Eq. 2 (Morton 1994).

### Statistical analyses

One-way analysis of variance was used to assess differences in power-duration parameters between models (Eqs. 1, 3, 4, and the best and worst individual fits), and for differences between the ${\dot{\text{V}}}$O_2peak_ achieved in the ramp incremental test and CWR prediction trials. Paired samples *t* tests and Bland–Altman analysis were used to evaluate differences between the actual and predicted T_lim_ for the ramp incremental tests. Pearson’s product moment correlation coefficient was used to assess relationships between the actual and predicted T_lim_ for the ramp incremental test, and the relationships between the error in estimation for the ramp incremental test T_lim_ and the CP, and Wʹ, respectively. Statistical significance was accepted at *P* < 0.05 and data are presented as mean ± SD.

## Results

The ${\dot{\text{V}}}$O_2peak_ measured during the ramp incremental test was 4.06 ± 0.60 L min^−1^ (54.7 ± 7.5 mL kg^−1^ min^−1^) and the peak work rate was 365 ± 57 W. The GET occurred at 2.19 ± 0.44 L min^−1^ and 141 ± 38 W. The ${\dot{\text{V}}}$O_2peak_ measured during the ramp incremental test was not different from the mean ${\dot{\text{V}}}$O_2peak_ in CWR prediction trials (4.05 ± 0.59 L min^−1^) measured at T_lim_ (*P* > 0.05).

There were no differences in CP or Wʹ estimates between the three models (i.e., Eqs. 1, 3, 4), or the best fit and the worst fit parameter estimates (*P* > 0.05; Table 1). The CP estimate from the best fit model corresponded to 66 ± 4% of the ramp incremental test peak power and 45 ± 6% Δ.

**Table 1:** The parameter estimates derived from Eqs. 1, 3 and 4, and the best (BIF) and worst individual fits (WIF). Total error indicates the sum of the coefficients of variation (CV %) associated with critical power (CP) and the curvature constant (Wʹ) of the power-duration relationship

	*R* ^2^	CP (W)	SEE (W)	CV %	Wʹ (kJ)	SEE (kJ)	CV %	Total error (CV %)
W–T_lim_ model	0.995–1.000	241 ± 48	3 ± 2	1.53 ± 1.22	18.6 ± 5.5	1.3 ± 0.8	7.6 ± 5.8	9.1 ± 6.9
1–T_lim_ model	0.931–1.000	242 ± 50	5 ± 3	2.10 ± 1.73	17.9 ± 4.4	1.2 ± 0.8	6.9 ± 4.9	8.9 ± 6.5
P–T_lim_ model	0.917–1.000	240 ± 48	3 ± 2	1.46 ± 1.29	18.5 ± 4.9	1.6 ± 1.3	9.2 ± 7.0	10.7 ± 8.1
BIF	0.969–1.000	242 ± 48	3 ± 2	1.33 ± 1.07	18.4 ± 5.7	1.0 ± 0.6	5.7 ± 4.2	7.3 ± 5.1
WIF	0.931–1.000	240 ± 50	5 ± 3	2.14 ± 1.84	18.5 ± 4.6	1.8 ± 1.3	9.8 ± 7.1	12.0 ± 8.5

*SEE* standard error of estimate, *T*_*lim*_ time to the limit of tolerance, *1/T*_*lim*_ linear inverse-of-time model, *P–T*_*lim*_ hyperbolic power-time model, *W–T*_*lim*_ linear work-time model

**Fig. 1:**
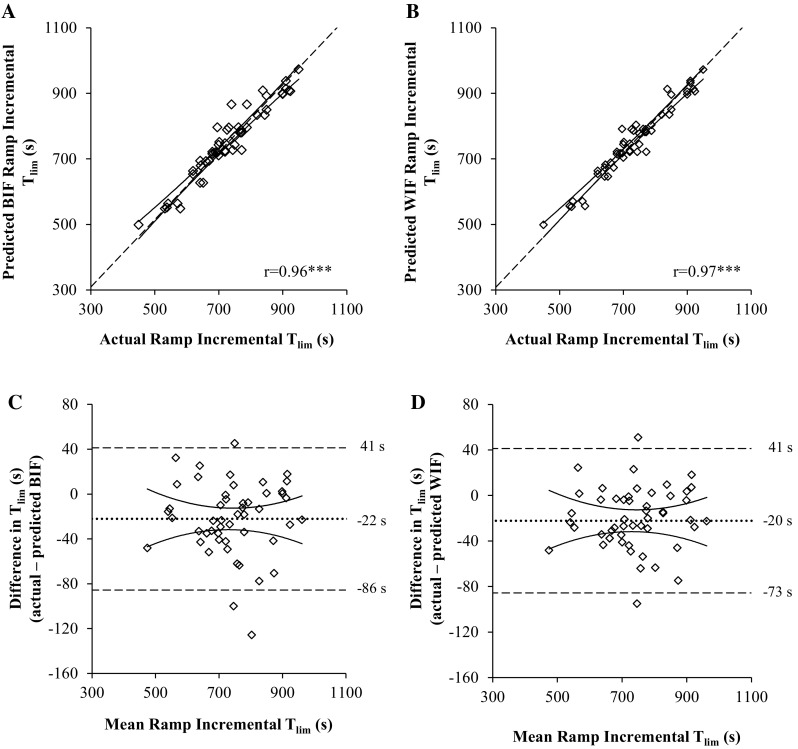
Bland-Altman plots of the relationship (**a** and **b**) and the limits of agreement (**c** and **d**) between the actual and predicted ramp incremental T_lim_ using the ‘best individual fit’ (BIF; **a** and **c**) and the ‘worst individual fit’ (WIF; **b** and **d**). **a** and **b** the *line* of origin (*dashed line*) and 95% confidence intervals (*solid lines*) are presented. **c** and **d**, the mean difference (*dotted line*), the 95% confidence intervals (*solid line*) and the limits of agreement (*dashed line*) are provided. ***P < 0.001

**Fig. 2:**
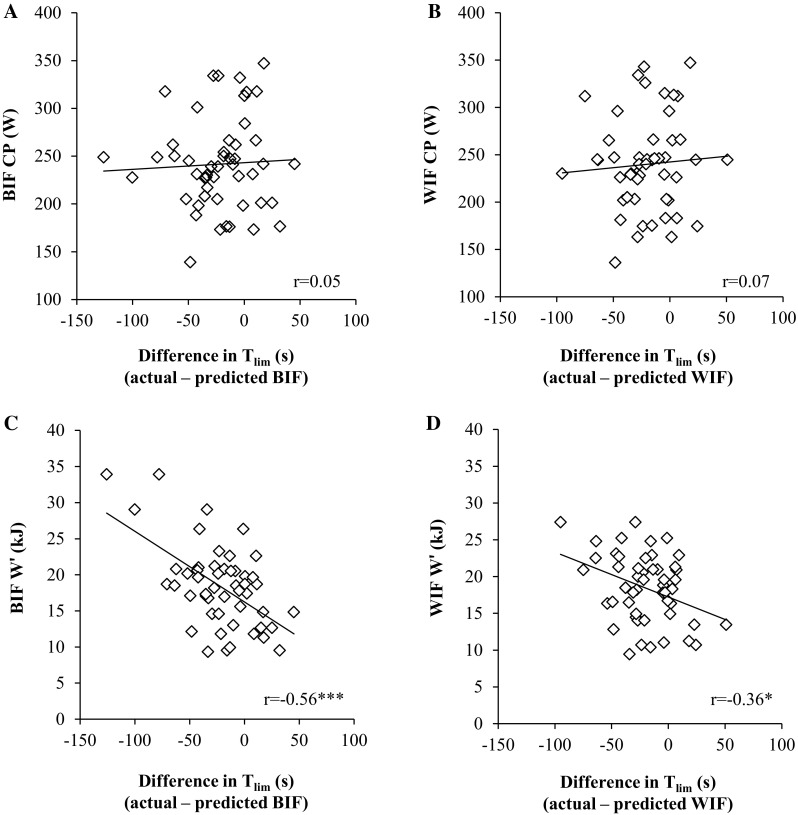
Relationship between the difference in actual and predicted T_lim_ derived from the ‘best individual fit’ (BIF; **a** and **c**) and the ‘worst individual fit’ (WIF; panels B and D) and the CP (**a** and **b**), and W′ (**c** and **d**). *P < 0.05, **P < 0.01, ***P < 0.001

The actual ramp incremental test T_lim_ (729 ± 113 s) was significantly correlated with the predicted T_lim_ calculated using the CP and Wʹ from the best fit model (751 ± 114 s, *r* = 0.96, *P* < 0.001) and the worst fit model (749 ± 111 s, *r* = 0.97, *P* < 0.001) (Fig. 1). However, both the best fit and worst fit models significantly overestimated T_lim_ with a mean bias of 22 s (CV 2.9 ± 2.4%) and 20 s (CV 2.6 ± 2.0%), respectively (Fig. 1). The error in the prediction was negatively correlated with the Wʹ from the best fit model (*r* = −0.56, *P* < 0.001) and the worst fit model (*r* = −0.36, *P* = 0.01), but was not significantly related to the CP (*P* > 0.05 for best and worst fit models) (Fig. 2).

## Discussion

The principal and novel findings of this study were that the CP and Wʹ derived from a series of CWR prediction trials significantly overestimated ramp incremental test performance. The overestimation in ramp incremental test performance was associated with the magnitude of the Wʹ, but not the CP. These findings may have important implications for normalisation of work rate in research settings, as well as for applied performance prediction, using the power-duration parameters derived from the conventional CWR prediction trial protocol.

In theory, when the CP and Wʹ are known, the power-duration relationship (Eqs. 1, 3, 4) should be applicable to predict performance in any severe intensity exercise bout irrespective of the work rate forcing function (Fukuba et al. 2003; Hill 1993; Jones et al. 2010; Morton 2006). To test this assumption, we performed a retrospective analysis of data sets for which the power-duration relationship had been estimated from a series of CWR prediction trials and used these parameter estimates to predict each subject’s ramp incremental exercise performance (Eq. 2; Morton 1994). The actual ramp incremental performance was overestimated by ~3% irrespective of whether the best or worst individual fits were used (Fig. 1). It should be noted that the coefficient of variation between the actual and predicted T_lim_ (~3%, or ~11 W) in the present study, consistent with previous data (CV %, 3 ± 3%, *n* = 7; Chidnok et al. 2013a), is fivefold greater than the typical test–retest reliability of a 30 W min^−1^ ramp incremental test performance (CV 0.53%; Weston and Gabbett 2001). This small, but consistent, overestimation in the performance prediction highlights the need for caution when using CP and Wʹ estimates derived from CWR protocols to predict exercise tolerance during ramp incremental exercise and potentially also during other work-rate forcing functions.

It has been previously shown that similar power-duration parameter estimates can be derived from two protocols employing contrasting work-rate forcing functions, that is: (1) a series of CWR trials, where the subject maintains a specified work rate for as long as possible; and (2) a 3 min all-out test, in which the subject exerts their maximal instantaneous power output throughout (Burnley et al. 2006; Simpson et al. 2015; Vanhatalo et al. 2007, 2008). Similarly, it has also been shown that the magnitude of the Wʹ is similar irrespective of its rate of utilisation (Fukuba et al. 2003; Chidnok et al. 2013a). It is important to note that in these experiments (Fukuba et al. 2003; Chidnok et al. 2013a) the Wʹ was estimated as the ‘work done >CP’, assuming that the CP itself was unaffected by different work rate forcing functions. Although the power-duration relationship was not established for the ramp incremental exercise in the present study, our findings suggest that there was a reduction in the CP and/or Wʹ during ramp incremental exercise relative to the CWR prediction trials. The only study to date that has directly compared the CP and Wʹ estimates derived from a series of CWR and ramp incremental prediction trials reported no difference in the CP but a tendency for a lower Wʹ during ramp incremental exercise (Morton et al. 1997). It is therefore likely that the overestimation of ramp incremental performance was due to a reduction in the Wʹ in ramp incremental exercise relative to the CWR protocol.

The mechanisms underlying a smaller Wʹ during ramp incremental exercise relative to CWR exercise may relate to differences in the motor unit recruitment patterns and ${\dot{\text{V}}}$O_2_ kinetics in response to different work rate forcing functions. The severe intensity CWR exercise trials necessitate a progressive increase in motor unit recruitment and/or firing frequency, which is consistent with an increase in integrated electromyography (iEMG) until T_lim_ (Vanhatalo et al. 2011). A similar increase in iEMG response is evident during ramp incremental exercise (Chidnok et al. 2013a; Scheuermann et al. 2002), but unlike CWR exercise, performance is dependent on the subjects’ ability to increase their work rate to meet the continually increasing, externally imposed work rate (e.g., 0.5 W s^−1^). There is some evidence to suggest that the accessible portion of the Wʹ may be partly determined by the rate of its utilisation and not merely by the capacity of Wʹ remaining (Chidnok et al. 2013b) The inability to achieve the higher imposed work rate, rather than task failure of motor units at a given constant work rate, may limit the accessible portion of the Wʹ, thus reducing ramp incremental exercise performance relative to that predicted from CWR prediction trials. In contrast, the power profile during the 3 min all-out test is not externally imposed but rather reflects the subject’s ability to generate maximal force which declines with time. Therefore, despite a reversal in the iEMG profile in the 3 min all-out test relative to CWR and ramp incremental exercise (i.e., a progressive decline in iEMG throughout the test) (Vanhatalo et al. 2011), it appears possible to access the Wʹ to the same extent during all-out and CWR severe intensity exercise (Simpson et al. 2015; Vanhatalo et al. 2007, 2008).

Although each subject attained a consistent ${\dot{\text{V}}}$O_2peak_ at T_lim_ following all experimental trials, the ${\dot{\text{V}}}$O_2_ kinetics differed significantly between protocols. During ramp incremental exercise, the ${\dot{\text{V}}}$O_2_ increases in proportion to the increase in work rate, displaying a quasi-linear response which persists even at work rates above the GET, at least during fast-ramp incremental protocols (Rossiter 2011; Whipp et al. 1981; Wilcox et al. 2016). In contrast, following an abrupt step increase to a constant work rate within the severe intensity domain (>CP), the ${\dot{\text{V}}}$O_2_ increases exponentially and is supplemented by an additional ${\dot{\text{V}}}$O_2_ slow component which elevates ${\dot{\text{V}}}$O_2_ to a greater value than that predicted from the extrapolation of ${\dot{\text{V}}}$O_2_ from work rates below the GET (Burnley and Jones^ 2007^; Rossiter 2011; Poole et al. 1988). Since the amplitude of the ${\dot{\text{V}}}$O_2_ slow component is positively correlated with the size of the Wʹ (Murgatroyd et al. 2011; Vanhatalo et al. 2011), it is possible that the overestimation of ramp incremental exercise performance by the CWR prediction trial protocol may be related to the limited scope for the development of the ${\dot{\text{V}}}$O_2_ slow component (and thus, incomplete access to Wʹ) during ramp incremental compared to CWR exercise. It may be speculated that accuracy of the ramp test performance prediction by the CWR prediction trial protocol may be improved by reducing the ramp rate considerably, thus revealing an upwardly curvilinear ${\dot{\text{V}}}$O_2_ response (Scheuermann et al. 2002).

An important observation in the present study was that the error in the ramp test performance prediction by Eq. 2 was correlated with the Wʹ, such that the greatest overestimation was evident in subjects with the largest Wʹ (Fig. 2). There was no relationship between the prediction error and the CP. These relationships provide further support for the interpretation that the accuracy of the ramp test performance prediction might have been adversely influenced by a discrepancy between the size of the Wʹ determined in a CWR protocol and the accessible portion of this Wʹ during ramp incremental exercise.

The close agreement between the parameter estimates derived from Eqs. 1, 3 and 4; the goodness of fit of each model to the experimental data; and the similarity of the CP estimates derived from the best (242 ± 48 W) and the worst (240 ± 50 W) individual fits (Table 1) manifest low incidence of random and systematic errors in the prediction trial data (Hill and Smith 1994). The CP and Wʹ estimates derived from the best and worst individual fits, therefore, predicted ramp test performance to a similar degree of (in)accuracy (Fig. 1). It should be noted, however, that the range of errors associated with the mathematical modelling of the Wʹ was considerably broader within the worst (CV % 0.11–34.4%) compared to the best individual fit (CV % 0.08–15.5%) (Table 1). Further research is warranted to identify whether the selection of the ‘best individual model fit’ for each subject is superior to conventional ‘one model fits all’ approach when predicting self-paced, maximal exercise performance that better reflects competitive sport.

In conclusion, ramp incremental exercise performance was not accurately predicted by the power-duration parameters derived from a series of CWR prediction trials. The parameter estimates overestimated actual performance. This overestimation was likely due to a reduction in the accessible portion of the Wʹ in the ramp test due to differences between the work-rate forcing functions and ${\dot{\text{V}}}$O_2_ kinetics in the two protocols (i.e., CWR vs. ramp incremental). This is consistent with the association between the predictive error and the magnitude of the Wʹ. Whilst it is recognised that ramp incremental exercise represents an extreme work-rate forcing function atypical of any sport, the inaccuracy in the prediction of ramp incremental performance highlights a potentially important consideration for the matching of prediction trials to the performance test. The present findings are consistent with the notion that the power-duration parameters are sensitive to interventions that alter ${\dot{\text{V}}}$O_2_ kinetics. Further investigation is warranted into effects of different work-rate forcing functions on the power-duration relationship when predicting exercise tolerance and performance in both research and applied settings.

## Abbreviations

Δ: Work rate difference between GET and the ${\dot{\text{V}}}$O_2peak_
CP: Critical power
CV %: Coefficient of variation
CWR: Constant work rate
GET: Gas exchange threshold
iEMG: Integrated electromyography
P: Power
*S*: Ramp slope
SEE: Standard error of estimate
τ: Time constant
T_lim_: Limit of tolerance
W: Work
$${\dot{\text{V}}}$$_E_: Minute ventilation
${\dot{\text{V}}}$CO_2_: Carbon dioxide output
$${\dot{\text{V}}}$$O_2_: Oxygen uptake
${\dot{\text{V}}}$O_2max_: Maximal oxygen uptake
${\dot{\text{V}}}$O_2peak_: Peak oxygen uptake
Wʹ: Curvature constant of the power-duration relationship
